# Developmental differences in affective representation between prefrontal and subcortical structures

**DOI:** 10.1093/scan/nsab093

**Published:** 2021-08-12

**Authors:** William J Mitchell, Lindsey J Tepfer, Nicole M Henninger, Susan B Perlman, Vishnu P Murty, Chelsea Helion

**Affiliations:** Department of Psychology, Weiss Hall, Temple University, Philadelphia, PA 19122, USA; Department of Psychological and Brain Sciences, Moore Hall, Dartmouth College, Hanover, NH 03755, USA; Klein College of Media and Communication, Annenberg Hall, Temple University, Philadelphia, PA 19122, USA; Department of Psychiatry, Washington University of St Louis, St Louis, MO 63110, USA; Department of Psychology, Weiss Hall, Temple University, Philadelphia, PA 19122, USA; Department of Psychology, Weiss Hall, Temple University, Philadelphia, PA 19122, USA

**Keywords:** affect, representational similarity analysis, development, ventromedial prefrontal cortex, subcortical region

## Abstract

Developmental studies have identified differences in prefrontal and subcortical affective structures between children and adults, which correspond with observed cognitive and behavioral maturations from relatively simplistic emotional experiences and expressions to more nuanced, complex ones. However, developmental changes in the neural representation of emotions have not yet been well explored. It stands to reason that adults and children may demonstrate observable differences in the representation of affect within key neurological structures implicated in affective cognition. Forty-five participants (25 children and 20 adults) passively viewed positive, negative and neutral clips from popular films while undergoing functional magnetic resonance imaging. Using representational similarity analysis to measure variability in neural pattern similarity, we found developmental differences between children and adults in the amygdala, nucleus accumbens and ventromedial prefrontal cortex (vmPFC): children generated less pattern similarity within subcortical structures relative to the vmPFC—a phenomenon not replicated among their older counterparts. Furthermore, children generated valence-specific differences in representational patterns across regions; these valence-specific patterns were not found in adults. These results may suggest that affective representations grow increasingly dissimilar over the course of development as individuals mature from visceral affective responses to more evaluative analyses.

## Introduction

A transition away from overt emotional reactivity is commonly observed over the course of early development (Karim and Perlman, 2017), and such age-related adaptations may be critical for successful functioning in the complex array of social and affective contexts that comprise adulthood (Camacho *et al.*, 2019). Some have argued that the emotional granularity of adults and children is comparable, pointing to research that has found children and adults reporting similar levels of emotional reactivity but differing in how they manage their responses when viewing affectively valenced stimuli (Silvers *et al.*, 2012). While individuals undoubtedly experience nuanced and powerful emotions throughout their life span, expressions of affectively relevant information change over development.

Children as young as 2 years use language to express emotions (Wellman *et al.*, 1995). However, the complexity of these expressions of emotion, as well as the recognition of others’ emotions (Fabes *et al.*, 1991), increases with age through early development. This development seems to be a function of social context, as family (Dunn *et al.*, 1991), peers (Fabes *et al.*, 1991) and language development (de Rosnay and Harris, 2002; Pons *et al.*, 2003; Nook *et al.*, 2017; Hoemann *et al.*, 2019) have an influence, with greater experiential variation resulting in greater individual differences. Researchers have postulated that these mediums function to improve mental representations of affective information (Pons *et al.*, 2003). Differentiation models of emotional development, such as those championed by Widen and Russell (2003, 2010), suggest that children begin with relatively simple, binarized categories, falling along lines of valence (i.e. positive/negative; Pons *et al.*, 2004; Widen, 2013) and that these categories progressively grow into more complex and multidimensional representations by adulthood (Russell, 1980, 2003; Widen and Russell, 2008; Nook *et al.*, 2017). Although by adulthood affective valence alone can be insufficient in explaining the physiological, expressive and experiential components of categorically congruent emotions (See Barrett, 2006, 2016), valence still demonstrates predictive utility in behaviors. For example, social norm violations of negative affect–related emotions elicit more punishment than those of positive affect–related emotions (Ansfield, 2007; Krumhuber and Manstead, 2009; Szczurek *et al.*, 2012). However, these signatures of differentiation in affective representations have primarily been documented via behaviors, leaving open questions about the underlying neural representations.

Previous studies have identified neural regions that undergo structural and functional changes in parallel with these cognitive and behavioral developments, including the ventromedial prefrontal cortex (vmPFC) (Gee *et al.*, 2013; Jalbrzikowski *et al.*, 2017), amygdala (AMY) (Davis and Whalen, 2001; Perlman and Pelphrey, 2011; Gabard-Durnam *et al.*, 2014), and nucleus accumbens (NAcc) (Levita *et al.*, 2009). In adults, the AMY and ventral striatum (VS), in which the NAcc is situated, may process the perception of valence and exert neuromodulatory influences on prefrontal circuitry (Davis and Whalen, 2001; Levita *et al.*, 2009). The vmPFC demonstrates a distinct pattern of outputs back to the AMY and NAcc, which may function as an affect and attention network (Bhanji *et al.*, 2019). However, the quality of the relationship between these structures may be different for young children and adults, as mPFC-AMY connectivity alters from positive to negative around the age of 10 years, with the valenced association in regional activation strengthening across normative development (Gee *et al.*, 2013). Furthermore, AMY-to-vmPFC projections emerge prior to vmPFC-to-AMY projections in rodents (Bouwmeester *et al.*, 2002a, b), which may offer a mechanism that explains behavioral studies finding that adults demonstrate greater emotional stability than children (Larson *et al.*, 1980; Noftle and Fleeson, 2010; Silvers *et al.*, 2017), as younger individuals may be at a deficit to modulate affective experiences via prefrontal–subcortical feedback loops. Late prefrontal development may be crucial to affective development in other ways, too, as the vmPFC has been tied to neural signatures of emotion classification in adults (Saarimäki *et al.*, 2016), which may support research finding that children have underdeveloped emotion differentiation skills relative to adults (Pons *et al.*, 2004). Changes in classification may actively shape neural representations within the mPFC, as well as the AMY and ventral anterior insula (Satpute *et al.*, 2016).

Although much is known about the functional and structural connections among these structures, how information is neurally represented is less well understood (Haxby *et al.*, 2014). Most of the investigations exploring neural mechanisms behind developmental changes in affectivity characterize the strength of activation (Camacho *et al.*, 2019), which may obscure more granular differences in how these stimuli are represented in the brain among adults and children (Popal *et al.*, 2019). For instance, if a univariate examination finds no difference between group responses when viewing affectively valenced stimuli, it is unclear whether granular and informative differences exist within neural representations, as any potential variations are subsumed when neural responses are averaged together. Techniques such as representational similarity analysis (RSA) (Kriegeskorte *et al.*, 2008) allow us to relate condition pairs using a metric that measures dissimilarity or similarity, depending upon the framing, between groups of correlative pairs and corresponding patterns of neural activity responding to each type of stimuli (Dimsdale-Zucker and Ranganath, 2018). Thus, the aim of this research is to further our understanding of developmental differences in affective representation using more modern methodology.

## Study goals and hypotheses

We applied an RSA approach to neuroimaging data collected from a sample of adults and children who viewed videos of both positive and negative affective social scenes from popular children’s movies, as well as neutrally valenced control stimuli, in order to determine whether developmental differences exist in affective representations. This was a secondary analysis of data originally collected by Karim and Perlman (2017), which solely investigated univariate activation and did not explore affective representations in the AMY, NAcc or vmPFC.

In line with developmental research suggesting the development of greater affective expressive and comprehension complexity with age, we hypothesized that children would generate greater pattern similarity relative to their older counterparts in response to valenced stimuli. Furthermore, due to the late functional development of the vmPFC, as well as the late development of projections from the mPFC region to subcortical structures, we expected that the difference in representational pattern similarity between children and adult subcortical regions of interest (ROIs) (AMY and NAcc) would be less than the difference between adults and children in the vmPFC. Understanding how children process representations of affectively valenced stimuli contributes not only to theories of neural processing but also provides practical knowledge of the developmental growth of higher-order thinking.

Finally, we expected that negatively valenced stimuli would generate greater representational similarity than positively valenced stimuli in both adults and children, due to evidence supporting the existence of negativity biases demonstrating reasonable consistency across individuals (Baumeister *et al.*, 2001). We also hypothesized a relative lack of differentiation, combined with the increased survival salience that negativity may signal (Pratto and John, 1991; O’Toole *et al.*, 2020), may result in this effect being more pronounced in children relative to adults.

## Materials and methods

### Participants

Fifty-seven English-speaking participants (36 children; 21 adults) with no history of psychiatric disorder were recruited at the University of Pittsburgh. All adult participants consented to study participation. All children assented to study participation and consent was provided by a parent. Twelve participants (11 children; 1 adult) were removed from analyses due to excessive head motion, resulting in an effective sample of 25 children (14 female, aged 4–10, *M* = 7.4, s.d. = 1.85) and 20 adults (9 female, aged 20–44, *M* = 26.7, s.d. = 5.20).

### Task

**Fig. 1.**
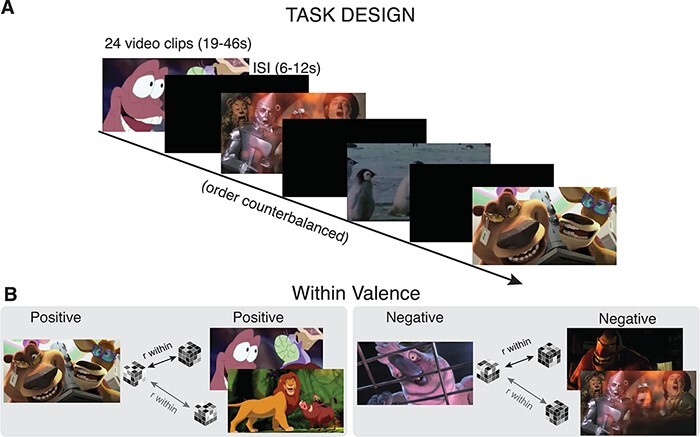
(A) Task design. In order to measure differences in representations, 20 adults (aged 20–44 years; mean age = 26.7 years; s.d. = 5.2 years) and 25 children (aged 4–10 years; mean age = 7.4 years, s.d. = 1.5 years) passively watched 24 positive, negative and neutral video clips from popular movies while undergoing an fMRI scan. Clips ranged from 19 to 46 s in length interspersed with a 6–12 s interstimulus interval. The order of the clips was counterbalanced across three conditions, and clips were standardized or matched across a number of metrics, including luminosity, valence intensity, duration by valence and familiarity. (B). Analysis. Using the data gathered during passive viewing of the stimuli, we used RSA to compute pairwise correlations within valence for positive, negative and neutral valenced stimuli. Correlative values were computed using the Spearman rank-order analysis for non-parametric data.

The task is explained in detail in Karim and Perlman (2017), but pertinent details will be highlighted here as well. Participants watched 24 clips from films while undergoing functional magnetic resonance imaging (fMRI) (Figure 1A and B). Those 24 clips were evenly divided into positive, negative, and neutral valence categories.

Positive and negative clips were sampled from popular children’s movies (e.g. classic movies such as *The Wizard of Oz* and Disney movies such as *Rio* and *Up*) such that each movie provided both a positive and negative clip for the stimuli set. Two films were live action (*The Wizard of Oz, Shark Boy and Lava Girl*), while the remaining six were animated (*Anastasia, Lion King, Little Mermaid, Open Season, Rio* and *Up*). The average amount of time participants spent viewing positive (253 s) and negative content (256 s) was approximately balanced. Positive and negative clips did not significantly differ in the total time [*t*(14) = −0.67, *P =* 0.51] or average time [*t*(14) = −0.67, *P =* 0.51] that faces were present on screen, so as to balance the presence of social stimuli between the two valenced conditions (Karim and Perlman, 2017). Affective development is closely associated with sociality, as social experiences and information are nearly ubiquitously cited in the emotional development literature as a medium by which children build upon their understanding of affective, and more specifically, emotional, representations (e.g. family unit: Dunn *et al.*, 1991, facial expressions: narratives: Pons *et al.*, 2003). Thus, utilizing film clips containing socially relevant information balanced between valenced conditions may represent a more ecologically appropriate means of motivating emotion representations than using stimuli devoid of social content.

Independent raters also coded the valence of each video second by second such that seconds of positive affect (e.g. smiles, cheering, etc.) received a score of 1, seconds of negative affect (e.g. anger, physical or verbal outbursts, etc.) received a score of −1 and seconds lacking a clearly valenced emotion (i.e. not containing elements of previously defined positive/negative cues) received a score of 0. As should be expected, positive and negative clips significantly differed in emotional valence [*t*(14) = 16.88, *P* < 0.001], with positive clips receiving an average second-by-second affective score of 0.89 and negative clips receiving an average affect score of −0.76. There were no significant differences in the absolute value of these scores by valence [*t*(14) = 1.38, *P =* 0.19], suggesting positive and negative clips were appropriately matched in time spent displaying categorically congruent valenced information (Karim and Perlman, 2017). Neutral films were sourced from nature documentaries, used animals or plants as the focal targets and included background music to mirror that of their affectively valenced counterparts. Participants were surveyed on their familiarity with each film and no differences were observed between adults and children in total familiarity scores [*t*(57) = −0.15, *P =* 0.89] or average familiarity scores [*t*(57) = 0.27, *P*  *=*0.79] (Karim and Perlman, 2017).

Individual film clips ranged from 19–46 s (*M *= 31.1 s) with a jittered black screen interstimulus interval (ISI) of 6–12 s, resulting in a total viewing time of 17 min (1020 s). To minimize the effects of emotional carryover, video clips were randomized into three orders and assigned to participants at random.

All participants completed a mock scanning session prior to data collection during which they were trained to remain motionless via a monitoring system that provided visual and auditory feedback. Participants also completed a short practice version of the task containing clips not used during primary data collection. During data collection, participants were reminded to remain motionless and asked to ‘watch the movies as they normally would’, with no further instructions provided. Exposure was followed by a short attention quiz, in which a single still frame was displayed and participants had to determine if it was pulled from a clip they just watched or a decoy image. Accuracy was sufficiently high for both children [*M* = 97.9%, s.d. = 3.3%] and adults [*M* = 92.9%, s.d. = 7.9%] (Karim and Perlman, 2017).

### Calculating inter-rater reliability of stimuli

Media content is often subjective, and, in confirming agreement on the constructs represented in the content, Krippendorff’s alpha (Lombard *et al.*, 2002; Krippendorff, 2004) is a common statistical comparison test used by media scholars to assess content constancy (Lombard *et al.*, 2002; Lombard, 2013). To assess inter-rater agreement of video category classification, three undergraduate research assistants (uninformed about this analysis or the hypotheses) coded the videos as either positive, negative or neutral in affective valence. Hayes’ SPSS Macro KALPHA was used to compute Krippendorff’s alpha (http://afhayes.com/spss-sas-and-r-macros-and-code.html) for inter-rater agreement of affective valence (*a* = 0.91 [CI: 0.83, 0.97]). Based on these criteria, the stimuli were deemed as constant in terms of the media representations of positive, negative and neutral stimuli categorization.

### Data acquisition

Data acquisition practices are outlined similarly in Karim and Perlman (2017). MRI images were obtained using a 3.0 T Siemens Trio scanner with a 12-channel parallel receive head coil. Structural images were obtained through a T-1 weighted MP-RAGE sequence where 175 sagittal (whole brain) slices were acquired. Functional whole brain blood oxygen level-dependent (BOLD) images were acquired in a sagittal left-to-right pattern, with the exception of a portion of the middle/superior temporal cortex within both hemispheres (Repetition Time (TR) = 2000 ms, Echo Time (TE) = 30 ms, flip angle (FA) = 90°, Field-of-view (FOV) = 256 mm, matrix size 64 × 64, voxel size 4 × 4 × 4 mm). Using a gradient echo echo-planar imaging sequence, 510 successive brain volumes were captured over 17 min and 6 s).

### Pre-processing

Structural and functional data were preprocessed to minimize the effects of head motion. Data were high-pass filtered using fMRI Expert Analysis Tool, and skull stripping was performed using Brain Extraction Tool) Both tools are included in FSL (v5.0; https://fsl.fmrib.ox.ac.uk) (Jenkinson *et al.*, 2012). Functional data were registered to anatomical images and nonlinearly warped to the MNI standard space. We identified head motion and noise-related factors by using timeseries data extracted from white matter and CSF, six head motion parameters, and their first derivatives to calculate and threshold metric values of how each time point was motion-affected. Additionally, individual TRs were identified and regressed out based on excessive head motion. Excessive head motion TRs were identified using the FSL Motion Outlier tool, which defines outlier thresholds as the 75th percentile plus 1.5 times the interquartile range. If more than 15% of TRs were considered outliers or if head motion values for any of the three rotations were greater than 1.5 mm, participants were excluded from analyses.

### Regions of interest

**Fig. 2.**
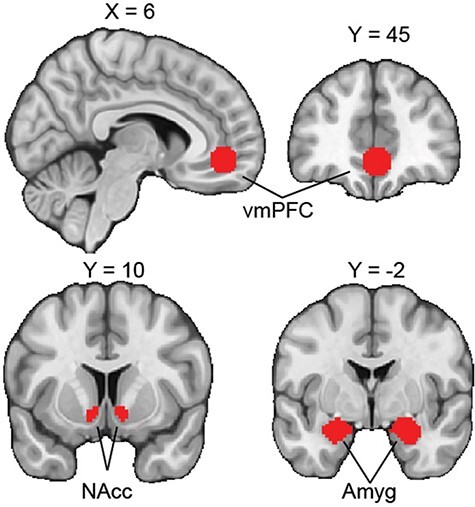
ROI masks. Our analysis focused on three regions identified by the extant literature as being pertinent to affective representations and which may demonstrate changes in representational patterns over early development into adulthood. These include the vmPFC, NAcc and amygdala. Amygdala and NAcc masks were taken from the Harvard–Oxford subcortical atlas, while the vmPFC was defined by a 10 mm diameter isotropic kernel centered on activation highlighted by Barta, McGuire, and Kable (2013).

We captured vmPFC data using an activation-centered mask with a 10 mm diameter isotropic kernel. Central MNI coordinates [X, Y, Z: 2, 46, −8] for the mask were identified in a meta-analysis by Bartra *et al.* (2013) as the most common center of consistent vmPFC activation during studies of subjective valuation and primary incentives. AMY and NAcc masks were taken from the Harvard–Oxford subcortical atlas (Frazier *et al.*, 2005; Makris *et al.*, 2006; Desikan *et al.*, 2006; Goldstein *et al.*, 2007). Masks were applied to all ROIs such that data from voxels beyond the bounds of the masks were excluded, and the included voxels were aligned with functional volumes. AMY and NAcc ROIs were thresholded at 50%. All masks were broadly defined in MNI space, applied to ROIs, and ROIs were transformed into subject native space using non-linear estimations (FNIRT tool in FSL). Transformations were visually inspected for accuracy. Mask placements are visualized in Figure 2.

### Statistical analyses

For each participant, we ran a general linear model (GLM) that had 24 regressors of interest, one for each positive, negative and neutral video clip. From these individual participant GLMs, we then extracted the activity for each voxel within each of our three ROIs (AMY, NAcc and vmPFC) for each of the video clips. The value of each voxel represents the average change in activation while passively viewing the video stimulus relative to baseline fixation cross measurements. To measure representational similarity within each of our three ROIs, the individual voxels contained within each participant’s GLM were aligned by MNI coordinates such that the same spaces were being compared to one another within-participant across clips.

Next, we used RSA to calculate our dependent measure. Pairwise complete observations of activity in each voxel within each ROI for each video clip were correlated with one another using the Spearman rank-order correlation method for non-parametric data. These correlation coefficients represent how similar the pattern of activation is within a given ROI between two different stimuli. In theory, the more similar the pattern of activation is, the more similarly the two stimuli are being represented by a neural structure (for a full review, see Popal *et al.*, 2019). Pairwise comparisons were performed across movie, but within valenced movie clips for each participant. For example, a positively valenced movie clip from *Lion King* was compared to a positively valenced movie clip from *The Little Mermaid.* This system produces an equal number of correlations in three within-valence categories: positive-to-positive comparisons, negative-to-negative comparisons and neutral-to-neutral comparisons. It is important to note that our positively valenced *Lion King* clip, for example, could *not* reliably be compared to *Lion King*’s own negatively valenced counterpart, as any two clips from the same source might demonstrate a high degree of representational similarity due to superficial or non-affective characteristics (e.g. common characters, audio motifs, stylistic overlap, etc.). This non-affective similarity would confound with any affective pattern similarity that was observed and would obscure the interpretation of our results. As such, only inter-movie comparisons were considered. Fisher’s Z-transformation was applied to all correlations before proceeding. Correlating the extracted GLM data from our 24 movie clips produced 28 correlative coefficients for each within-valence comparison per participant per ROI.

We were primarily concerned with exploring three effects: (i) the interaction of age and ROI such that adults and children may show greater similarity to one another in subcortical affective representation relative to vmPFC representation, (ii) a contrast of valence within age group or whether children demonstrate measurably greater representational similarity relative to adults towards valenced stimuli (i.e. positive and negative) and (iii) the interaction of age and valence or whether we’d find that children demonstrate a greater discrepancy in negative and positive affective representational similarity than do adults.

To accurately represent age group as a between subject fixed effect and ROI and valence category as within subject fixed effects, data were analyzed using a 3 (ROI: AMY, NAcc, vmPFC) × 2 (age group: children, adults)× 3 (valence type: positive, negative, neutral) mixed effects Analysis of Variance (ANOVA). Results from our ANOVA were followed with Bonferroni-adjusted post-hoc contrasts to further elucidate the relationship among our effects. Analyses were performed using the R statistical programming language (v4.0.3; http://www.R-project.org/) in conjunction with the Integrated Development Environment, RStudio (v1.3.1093; https://rstudio.com/).

Due to the extensively documented neurodevelopmental changes children experience in our age range (4 –10 years), there was some concern that while convenient, categorizing participants ages 4–10 into the same developmental category may conceal important variability within the samples (Gee *et al.*, 2013). As a result, an additional analysis was performed to determine whether age predicted representational similarity values treating our Fisher’s Z-transformed correlation values as a criterion variable in a multilevel model predicted by the fixed effects of age, measured as a continuous variable in months, and participant, as a random effect, with random intercepts and fixed slopes.

## Results

Using multilevel regression, we failed to find predictive utility for age in months towards representational similarity among our child sample (β = 0.085, se = 0.143, *P* > 0.05), lending further support to the categorical boundaries we had defined. This model failed to outperform a null model lacking fixed effects (ICC = 0.094).

**Fig. 3.**
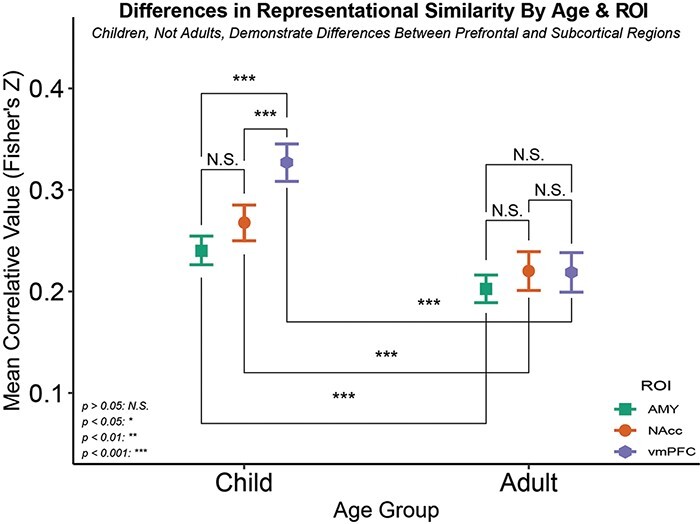
Differences in representational similarity by age group and ROI. After pre-processing, data from every voxel in each of our three ROIs were aligned by MNI coordinates such that the same spaces were being compared to one another within-participant across clips. We then ran Spearman rank-order correlations across all pairwise complete observations and Fisher’s Z transformation. These correlation coefficients represent how similar the pattern of activation is within a given ROI of a participant between two different stimuli, or representational similarity. These values were then entered into a 3 (ROI: AMY, NAcc, vmPFC) × 2 (age group: children, adults) × 3 (valence type: positive, negative, neutral) mixed effects ANOVA. No differences were found in representational similarity between Adult AMY, NAcc and vmPFC. Children’s AMY and NAcc differed from vmPFC in representational similarity such that different stimuli of the same valence showed more similar patterns of activation within the vmPFC than in subcortical regions. Children demonstrated significantly greater pattern similarity across all ROIs than their adult counterparts. This supports behavioral and cognitive research suggesting greater differentiation in affective processing following connectivity developments over these developmental periods, which are most pronounced in prefrontal regions.

**Note**: As a result of our Fisher’s Z transformation, our ‘Mean Correlative Value’ could range from −1.77–1.76. The range has been restricted in this visualization for the sake of comprehending differences among groups. Error bars represent 95% confidence intervals.

Using a mixed effects ANOVA model adjusting for the random effect of participant, significant differences were measured in the interaction between ROI and age group [*F*(2, 11 279) = 10.13, *P* < 0.001]. Bonferroni-adjusted post-hoc contrasts further illustrated that children demonstrated greater representational similarity in AMY [*t*(3757) = 3.784, *P*_adj._* *< 0.001], NAcc [*t*(3638) = 3.588, *P*_adj._* *< 0.001] and vmPFC [*t*(3676) = 7.901, *P*_adj._* *< 0.001] activation patterns relative to adults (Figure 3). Children also demonstrated differences in representational similarity between the AMY and vmPFC [*t*(3931) = −7.300, *P*_adj._* *< 0.001] and NAcc and vmPFC [*t*(4188) = −4.569, *P*_adj._* *< 0.001], but not AMY and NAcc [*t*(4013) = −2.361, *P*_adj._ = 0.468]. No such ROI differences were observed in our adult sample [AMY-NAcc: *t*(3036) = −1.465, *P*_adj._ = 1.000; AMY-vmPFC: *t*(3004) = −1.341, *P*_adj._ = 1.000; NAcc-vmPFC: *t*(3357) = 0.092, *P*_adj._ = 1.000]. Interaction contrasts found the differences between the adult AMY and vmPFC to be different from that of the child AMY and vmPFC [*t*(7551) = −4.766, *P*_adj._* *< 0.001], as well as between vmPFC and NAcc, [*t*(7553) = 3.511, *P*_adj._ < 0.001], but no such differences were observed between the AMY and NAcc [*t*(7558) = −0.875, *P*_adj._ = 1.000]. See Table 1 for additional ROI and age group contrast results.

**Table 1.** Bonferroni-adjusted age group and ROI contrast results

Contrasts	Mean difference	SE	*t*-Statistic	df
AMY, child *vs* adult	0.037	0.010	3.784***	3757
NAcc, child *vs* adult	0.048	0.013	3.588***	3638
vmPFC, child *vs* adult	0.108	0.014	7.901***	3676
child, AMY *vs* NAcc	−0.028	0.011	−2.361	4013
child, AMY *vs* vmPFC	−0.087	0.012	−7.300***	3931
child, NAcc *vs* vmPFC	−0.059	0.013	−4.569***	4188
adult, AMY *vs* NAcc	−0.017	0.012	−1.465	3036
Adult, AMY *vs* vmPFC	−0.016	0.012	−1.341	3004
Adult, NAcc *vs* vmPFC	0.001	0.014	0.092	3357
AMY, child and NAcc, adult *vs* AMY, adult and NAcc, child	−0.008	0.008	−0.875	7558
AMY, child and vmPFC, adult *vs* AMY, adult and vmPFC, child	−0.041	0.009	−4.766***	7551
NAcc, child and vmPFC, adult *vs* NAcc, adult and vmPFC, child	0.033	0.010	3.511***	7553

Note: All significance values were bonferroni-adjusted; p < 0.05: * ; p < 0.01: ** ; p < 0.001: ***.

**Fig. 4.**
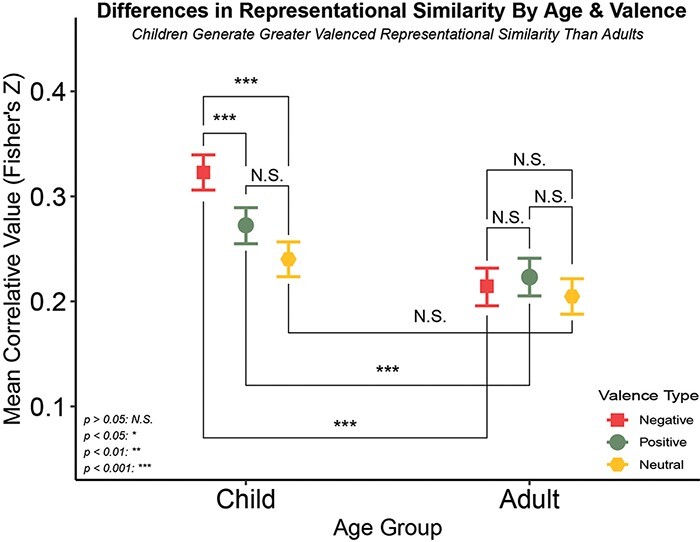
Differences in representational similarity by age group and ROI. After pre-processing, data from every voxel in each of our three ROIs were aligned by MNI coordinates such that the same spaces were being compared to one another within-participant across clips. We then ran Spearman rank-order correlations across all pairwise complete observations and Fisher’s Z transformed. These correlation coefficients represent how similar the pattern of activation is within a given ROI of a participant between two different stimuli or representational similarity. These values were then entered into a 3 (ROI: AMY, NAcc, vmPFC) × 2 (age group: children, adults) × 3 (valence type: positive, negative, neutral) mixed effects ANOVA. Adults demonstrated no differences in representational similarity by valence. However, children demonstrated greater similarity to negative stimuli relative to positive and neutral stimuli, but not between positive and neutral stimuli. Age differences were significant between positive and negative, but not neutral stimuli. Developmental decreases in representational pattern similarity into adulthood may support constructivist theories of emotional development, suggesting the development of greater nuance over time.

**Note**: As a result of our Fisher’s Z transformation, our ‘Mean Correlative Value’ could range from −1.77–1.76. The range has been restricted in this visualization for the sake of comprehending differences among groups. Error bars represent 95% confidence intervals.

Contrasts of valence within age group suggest that adults do demonstrate relatively less representational similarity in response to valenced stimuli [*t*(7355) = −8.863, *P*_adj._* *< 0.001] compared to their younger counterparts (Figure 4). The interaction between age group and valence was also deemed significant [*F*(2, 11 279) = 10.74, *P* < 0.001] with children showing greater positive [*t*(3687) = 3.872, *P*_adj._* *< 0.001] and negative [*t*(3660) = 8.693, *P*_adj._* *< 0.001], but not neutral [*t*(3710) = 2.938, *P*_adj._ = 0.078], representational similarity relative to adults. Similar to ROI, adults did not differentiate among any valence categories [Pos-Neg: *t*(3358) = 0.727, *P*_adj._ = 1.000; Pos-Neut: *t*(3347) = 1.470, *P*_adj._ = 1.000; Neg-Neut: *t*(3346) = 0.720, *P*_adj._ = 1.000]. Children, however, did show greater similarity for negative affective stimuli over both their positive [*t*(4196) = −4.142, *P*_adj._ < 0.001] and neutral [*t*(4197) = 6.882, *P*_adj._ < 0.001] counterparts. Positive affective stimuli did not show greater similarity above that of neutral stimuli in children [*t*(4192) = 2.637, *P*_adj._ = 0.208]. The difference between valenced and non-valenced stimuli was significant in children [*t*(4306) = 5.504, *P*_adj._ < 0.001], but not adults [*t*(3542) = 1.277, *P*_adj._ = 1.000]. See Table 2 for additional valence and age group contrast results, and Table 3 for ANOVA results.

**Table 2.** Bonferroni-adjusted age group and valence contrast results

Contrasts	Mean difference	SE	*t*-Statistic	df
Positive, child *vs* adult	0.049	0.013	3.872	3687
Negative, child *vs* adult	0.109	0.013	8.693	3660
Neutral, child *vs* adult	0.035	0.012	2.938	3710
Child, positive *vs* negative	−0.051	0.012	−4.142	4196
Child, positive *vs* neutral	0.032	0.012	2.637	4192
Child, negative *vs* neutral	0.083	0.012	6.882***	4197
Adult, positive *vs* negative	0.009	0.013	0.727	3358
Adult, positive *vs* neutral	0.018	0.013	1.470	3347
Adult, negative *vs* neutral	0.009	0.013	0.720	3346
Positive, child and negative, adult *vs* positive, adult and negative child	0.032	0.009	3.613***	7558
Child, valenced *vs* neutral	0.057	0.010	5.504***	4306
Adult, valenced *vs* neutral	0.013	0.011	1.277	3542
Valenced, child and neutral, adult *vs* valenced, adult and neutral, child	0.044	0.007	6.131***	11306
Valenced, child *vs* valenced, adult	0.079	0.009	8.863***	7355

Note: All significance values were bonferroni-adjusted; p < 0.05: * ; p < 0.01: ** ; p < 0.001: ***.

**Table 3.** Mixed effects ANOVA results

Variable	Sum of squares	*df*	Mean square	*F*	*P*	Partial ω^2^	Cohen’s *F*
Error: Participant							
Age group	11.6	1	11.63	3.56	0.066	0.008	0.088
Residuals	140.3	43	3.26				
Error: Within							
ROI	6.0	2	2.91	21.84	<0.001	0.004	0.062
Valence	5.0	2	2.36	17.69	<0.001	0.003	0.056
ROI × age group	3.0	2	1.35	10.13	<0.001	0.002	0.042
ROI × valence	0.0	4	0.11	0.79	0.530	0.000	0.017
Age group × valence	3.0	2	1.43	10.74	<0.001	0.002	0.044
Age group × ROI × valence	0.0	4	0.06	0.45	0.770	0.000	0.013
Residuals	1504.0	11 279	0.13				

## Discussion

This study represents a first attempt to compare developmental differences between adults and children in prefrontal and subcortical regions concerning cognitive representations of affective information. Based on extant literature, we expected children to demonstrate a greater discrepancy in pattern activation between the vmPFC and subcortical structures relative to adults due to the literature suggesting the late development of projections from the vmPFC to subcortical affective structures and greater emotional granularity with age. Between childhood and early adulthood, we witnessed a marked decrease in affectively valenced pattern similarity. Furthermore, valence-specific differences and region-specific differences that were present in children, having demonstrated greater similarity for negative stimuli compared to positive and within the vmPFC relative to subcortical structures (AMY and NAcc), were absent in our adult sample. Taken together, these results may suggest that affective representations decrease in pattern similarity over normative development although longitudinal designs may be better suited to demonstrate a casual trajectory. With an emphasis on the notable representational differences in vmPFC development, we interpret these results to suggest that people may experience a maturation from visceral affective responses that merely assess how evocative an affective experience is, to more evaluative analyses which modulate affective responses between childhood and adulthood. This supposition is supplemented by extant findings from the neurobehavioral literature.

For example, activation of the AMY has demonstrated a consistent positive association with affective physiological markers, such as heart rate variability (Wei *et al.*, 2018) and perspiration (Asahina *et al.*, 2003), as well as reports of affective intensity (Bonnet *et al.*, 2015). Activity changes in the NAcc have coincided with reports of valenced affective intensity, as well (Knutson and Greer, 2008). For both the VS and AMY, the extent to which their affective contributions can be modulated seems to be a consequence of their functional connectivity to medial prefrontal regions (Cohen *et al.*, 2009; Sakaki *et al.*, 2016; Wei *et al.*, 2018). Univariate methods also support potential differences in NAcc contributions to affect processing across age, and the increased risk of depression upon alterations in NAcc activity emphasizes the importance of this region for adequate emotional development (Monk *et al.*, 2008). In addition to univariate interpretations, multivariate studies have disentangled discrete emotional categories from patterns of activity across several neural regions (Saarimäki *et al.*, 2016; Camacho *et al.*, 2019) and, more specifically, within the vmPFC (Kragel and LaBar, 2015, 2016). When disentangling multivariate patterns for discrete emotional categories, the vmPFC, in addition to the inferior lateral PFC, contributed most to how well the neural signals could classify each emotion (Saarimäki *et al.*, 2016), resonating with an established body of work tying the vmPFC to emotion (Greene, 2007; Moll and de Oliveira-souza, 2007; Winecoff *et al.*, 2013; Hiser and Koenigs, 2018; Yang *et al.*, 2018). As such, relatively late neurodevelopmental changes in the mPFC, including the development of projections to affective structures like the AMA and NAcc, may be a cruical component in explaining developmental divergences in affective representations.

While the vmPFC likely performs some affective processing in and of itself, it may also regulate affective processing in the NAcc and AMY (Hiser and Koenigs, 2018). The development of functional connectivity between the medial prefrontal cortex (mPFC) and AMY (Bouwmeester *et al.*, 2002a; Gee *et al.*, 2013) may also contribute to greater control over these physiological and experiential affective responses, which may also contribute to a greater variety, allowing for greater nuance, in affective representation. Perhaps, as suggested by others (Larson *et al.*, 1980; Noftle and Fleeson, 2010; Silvers *et al.*, 2017), younger individuals may be at a deficit to modulate affective experiences via prefrontal–subcortical feedback loops.

Our results finding age specificity in pattern similarity is in line with developmental perspectives of the vmPFC’s role in emotional processing and aligns with past work demonstrating distinctions between this area’s function across age. For instance, Silvers *et al.* (2017) found age-related decreases in vmPFC response to emotional photos, with larger degrees of vmPFC involvement found among younger participants relative to older ones. While mPFC-VS resting state functional connectivity demonstrates a positive relationship from childhood through early adulthood, whether connectivity increases with age (Di Martino *et al.*, 2008, 2011) or remains stable through development (Greene *et al.*, 2014; Fareri *et al.*, 2015) is debated. The magnitude of task-based functional connectivity between mPFC and VS also appears to be variable dependent upon the task-based context (Richards *et al.*, 2013) although age-related linear increases have been observed for positive incentives (van den Bos *et al.*, 2012). Regardless of potential changes in connectivity, it may be the case that the connection between VS and vmPFC serves a stimulus evaluation role (Salzman *et al.*, 2007; Bartra *et al.*, 2013; Fareri *et al.*, 2015) that may modulate value-related signals relevant to affectively valenced stimuli. Taken together, a potential interpretation of our findings is that, in light of the region’s role in processing of emotional stimuli, the pattern and response similarities we find in children may signal their shared, relatively limited experience of the vast array of potential affectively relevant experiences, while adults respond more divergently to novel affectively valenced information by referencing a wider berth of past evaluations. Our study connects findings from behavioral affective development and neural representations of affect by demonstrating that observed developmental behavioral differences between adults and children extend to neural representations of affective information within the vmPFC, NAcc and AMY specifically.

The cognitive literature also presents at least a few mechanisms by which representations may grow more dissimilar with age. Appraisal theories of emotion broadly posit that emotions are elicited by or are emergent phenomena from evaluations of events and circumstances (Ortony *et al.*, 1988; Roseman and Smith, 2001; Clore and Ortony, 2008). These evaluations may be colored by the biases and information individuals already possess. It may be the case that the greater potential for experiential variance inherent to having had lived longer may add nuance or variation to adult representations relative to children. Similar mechanisms have been theorized by other researchers (e.g. Pons *et al.*, 2003), and this postulation fits nicely with rational constructivist-related theories of emotional development, in which humans start with proto-conceptual primitives to emotion that mature over time due to language and symbol learning, Bayesian inductive learning and constructive thinking mechanisms (Hoemann *et al.*, 2019; for a review, see Fedyk and Xu, 2018). Relatedly, our ability to consider the multidimensionality of affect-related concepts improves with age as well, which could logically lead to greater idiosyncratic processing. This may speak to our age-related valence findings, as adults likely defaulted to representing emotional information continuously, rather than strictly categorically (see Satpute *et al.*, 2016). This is conceptually consistent with work from Nook *et al.* (2017, 2018) as they had found that affective complexities like valence and arousal change as a function of age. As such, our conceptions of affective experiences may evolve over development from notions relatively faithful to valence-general lines (i.e. ‘good’, ‘bad’) to representing a more nuanced, multidimensional understanding of affective experiences.

While adults did demonstrate lower levels of similarity in response to valence than children, it is notable that the correlation was not non-existent. As a result, valence likely still plays a role in shaping some aspects of decision-making and behavior and may explain the findings like those suggesting greater homogeneity in response to norm violations of negative affect than positive affect, with targets who express positive or neutral responses to negatively perceived stimuli to be judged as less authentic (Krumhuber and Manstead, 2009), socially appropriate and less liked (Ansfield, 2007), as well as punished more harshly than incongruent responses to differently valenced stimuli (Szczurek, Monin, and Gross, 2012). These findings support previous suppositions regarding an information value asymmetry between positive and negative affective experiences, as negative information may signal a need for adjustment to avoid detrimental consequences (Pratto and John, 1991; O’Toole *et al.*, 2020).

Although we can provide our interpretation of the data, we possess neither the statistical resolution nor the appropriate research design to write with absolute certainty what mechanism or mechanisms may be at play here. Work from Brooks and Freeman (2018) does suggest coherence between self-reported conceptual similarity and representational similarity in emotion perception, which supports claims connecting RSA findings to ecologically valid phenomena; however, future work relating differences in representational similarity to the accrual and modeling of affective experience is needed.

### Study limitations and future directions

First, some logistical concerns relevant to human neuroimaging in a developmental population must be highlighted. Our effective sample size of 25 children and 20 adults is small for a study spanning such a large age range. Related to our limited sample is the disproportionately greater frequency of head motion artifacts commonly produced when scanning children (Greene *et al.*, 2016), which resulted in 11 children being excluded from our analyses (as compared to only 1 adult). There is some concern regarding comparability of neural structures and responses over development. Children do demonstrate greater BOLD signal response both at rest and during neural activity relative to adolescents and adults although this difference is likely non-significant following normalization (Moses *et al.*, 2014; Bray, 2017). Additionally, due to structural maturations through development, the use neural atlases not specialized for children (e.g. Harvard Oxford) may depreciate in utility although the extent is unclear.

While our results suggest a number of differences in affective representation between children and adults, it is difficult to conclude with certainty exactly what implications this similarity has for cognition and behavior without additional measurements. Although we can make inferences, our RSA analysis is incapable of commenting with any certainty as to the contents of representations or in what ways they are similar or dissimilar. Our interpretations of these results are based upon the extant literature exploring the functions of the AMY, NAcc and vmPFC in similar experimental contexts, but the debate as to the precise functions of each is still widely contested. For example, meta-analyses suggest that the posterior vmPFC, for example, differentially responds to emotion, while the rostral and central vmPFC demonstrate increased activation during social processing and valuation judgments, respectively (Hiser and Koenigs, 2018), which may suggest a need for more precise anatomically defined masking to explore questions of affectivity in the vmPFC.

To address concerns that observed patterns may be in response to non-affective characteristics of the stimuli, we compared the pattern similarity of affectively valenced video pairs to pairwise comparisons of our neutral stimuli set. Representational similarity was greater for affective stimuli than non-affective stimuli in children [*t*(4306) = 5.504, *P*_adj._ < 0.001] but not adults [*t*(3542) = 1.277, *P*_adj._ = 1.000]. This was surprising, as it could suggest valence to be too simple of a metric by which to classify neural activation patterns in adults. However, it is more likely that there were characteristics of our neutral video set that limit their utility as ideal controls. While valenced stimuli consisted of both animated and live-action videos, our neutral videos were strictly live-action videos, which may have influenced how participants evaluated them. Similarly, the presence of socially relevant stimuli (i.e. use of language, humans and anthropomorphized animals) was not balanced between valenced and neutral videos. Additionally, while categorical coherence was assessed twice for valenced videos and once for neutral videos by independent raters, the subjective evaluations or interpretations of individual participants in response to the stimuli may have varied more than anticipated. This could be especially problematic with such a small dataset. As such, any differences observed between valenced and non-valenced (i.e. neutral) representations should especially be skeptically considered in the absence of replication with more suitable controls.

An additional limitation is the lack of resolution of emotional classifications among our affective observations. Although affective representations like those we had focused on this study may relate to representations of emotions, they are not synonymous, and emotions that might typically be considered categorically congruent in terms of affect can vary dramatically in terms of expression, experience and physiology. For example, studies that have compared activation patterns of different ‘negatively valenced’ emotions have found dramatic differences, especially within the basal ganglia and medial PFC (Kassam *et al.*, 2013). Furthermore, expression, experience and physiology of the same emotions can vary dramatically from person to person (see Clore and Ortony, 2013) and culture to culture (Ma *et al.*, 2018). Our investigation lacked the statistical power to analyze affective classifications at a more granular level (i.e. emotions), and, as such, is limited in applicability.

## Conclusions

The present study found differences in how similarly children and adults represent affective stimuli within the AMY, NAcc and vmPFC, as well as differences by valence. The representation of affect in subcortical structures, like the AMY and NAcc, may change relatively less across the life span than frontal regions, such as the vmPFC, which may be indicative of a maturation from passive assessment to active engagement with or modulation of affectively relevant experiences. Although it may be natural as an adult to pine for the relative simplicity with which we assessed our childhood experiences, our findings suggest these might be necessary trade-offs in the development of mature, nuanced understandings of our emotional experiences.

## Data Availability

This research data, masks, stimuli and associated scripts have been made available at https://osf.io/9nhwt/.

## References

[R1] Ansfield M.E. (2007). Smiling when distressed: when a smile is a frown turned upside down. *Personality & Social Psychology Bulletin*, 33(6), 763–75. 17483396 10.1177/0146167206297398

[R2] Asahina M., Suzuki A., Mori M., Kanesaka T., Hattori T. (2003). Emotional sweating response in a patient with bilateral amygdala damage. *International Journal of Psychophysiology*, 47(1), 87–93. 12543449 10.1016/s0167-8760(02)00123-x

[R3] Barrett L.F. (2006). Solving the emotion paradox: categorization and the experience of emotion. *Personality and Social Psychology Review*, 10(1), 20–46. 16430327 10.1207/s15327957pspr1001_2

[R4] Barrett L.F. (2016). The theory of constructed emotion: an active inference account of interoception and categorization. *Social Cognitive and Affective Neuroscience*, 12(1), 1–23. 10.1093/scan/nsw154 PMC5390700 27798257

[R5] Bartra O., McGuire J.T., Kable J.W. (2013). The valuation system: a coordinate-based meta-analysis of BOLD fMRI experiments examining neural correlates of subjective value. *Neuroimage*, 76, 412–27. 23507394 10.1016/j.neuroimage.2013.02.063 PMC3756836

[R6] Baumeister R.F., Bratslavsky E., Finkenauer C., Vohs K.D. (2001). Bad is stronger than good. *Review of General Psychology*, 5(4), 323–70.

[R7] Bhanji J., Smith D.V., Delgado M. (2019). A brief anatomical sketch of human ventromedial prefrontal cortex [preprint]. PsyArXiv. doi: 10.31234/osf.io/zdt7f

[R8] Bonnet L., Comte A., Tatu L., Millot J.-L., Moulin T., Medeiros de Bustos E. (2015). The role of the amygdala in the perception of positive emotions: an “intensity detector”. *Frontiers in Behavioral Neuroscience*, 9, 1–12. doi: 10.3389/fnbeh.2015.00178 PMC4493392 26217205

[R9] Bouwmeester H., Smits K., Van Ree J.M. (2002a). Neonatal development of projections to the basolateral amygdala from prefrontal and thalamic structures in rat. *The Journal of Comparative Neurology*, 450(3), 241–55. 12209853 10.1002/cne.10321

[R10] Bouwmeester H., Wolterink G., Van Ree J.M. (2002b). Neonatal development of projections from the basolateral amygdala to prefrontal, striatal, and thalamic structures in the rat. *The Journal of Comparative Neurology*, 442(3), 239–49. 11774339 10.1002/cne.10084

[R11] Bray S. (2017). Age-associated patterns in gray matter volume, cerebral perfusion and BOLD oscillations in children and adolescents: multimodal imaging of brain development. *Human Brain Mapping*, 38(5), 2398–407. 28117505 10.1002/hbm.23526 PMC6866757

[R12] Brooks J.A., Freeman J.B. (2018). Conceptual knowledge predicts the representational structure of facial emotion perception. *Nature Human Behaviour*, 2(8), 581–91. 10.1038/s41562-018-0376-6 PMC6788630 31209318

[R13] Camacho M.C., Karim H.T., Perlman S.B. (2019). Neural architecture supporting active emotion processing in children: a multivariate approach. *Neuroimage*, 188, 171–80. 30537564 10.1016/j.neuroimage.2018.12.013 PMC6401267

[R14] Clore G.L., & Ortony A. (2008). Appraisal theories: How cognition shapes affect into emotion. In: Lewis, M., Haviland-Jones, J. M., & Barrett, L. F., editors. Handbook of emotions, 3rd ed. pp. 628–42, New York, NY: The Guilford Press.

[R15] Clore G.L., & Ortony A. (2013). Psychological construction in the OCC model of emotion. *Emotion Review*, 5(4), 335–43. 25431620 10.1177/1754073913489751 PMC4243519

[R16] Cohen M.X., Schoene-Bake J.-C., Elger C.E., Weber B. (2009). Connectivity-based segregation of the human striatum predicts personality characteristics. *Nature Neuroscience*, 12(1), 32–4. 19029888 10.1038/nn.2228

[R17] Davis M., Whalen P.J. (2001). The amygdala: vigilance and emotion. *Molecular Psychiatry*, 6(1), 13–34. 11244481 10.1038/sj.mp.4000812

[R18] Desikan R.S., Fischl B., Quinn B.T., et al. (2006). An automated labeling system for subdividing the human cerebral cortex on MRI scans into gyral based regions of interest. *Neuroimage*, 31(3), 968–80. 16530430 10.1016/j.neuroimage.2006.01.021

[R19] Di Martino A., Scheres A., Margulies D.S., et al. (2008). Functional connectivity of human striatum: a resting state fMRI study. *Cerebral Cortex*, 18(12), 2735–47. 18400794 10.1093/cercor/bhn041

[R20] Di Martino A., Kelly C., Grzadzinski R., et al. (2011). Aberrant striatal functional connectivity in children with autism. *Biological Psychiatry*, 69(9), 847–56. 21195388 10.1016/j.biopsych.2010.10.029 PMC3091619

[R21] Dimsdale-Zucker H.R. Ranganath C. (2018). Chapter 27 - Representational similarity analyses: a practical guide for functional MRI applications. In: Manahan-Vaughan, D., editor. *Handbook of Behavioral Neuroscience*, Vol. 28, pp. 509–25, Elsevier. doi: 10.1016/B978-0-12-812028-6.00027-6

[R22] Dunn J., Brown J., Beardsall L. (1991). Family talk about feeling states and children’s later understanding of others’ emotions. *Developmental Psychology*, 27(3), 448–55.

[R23] Fabes R.A., Eisenberg N., Nyman M., Michealieu Q. (1991). Children’s appraisals of others’ spontaneous emotional reactions. *Developmental Psychology*, 27(5), 858–66.

[R24] Fareri D.S., Gabard-Durnam L., Goff B., et al. (2015). Normative development of ventral striatal resting state connectivity in humans. *Neuro Image*, 118, 422–37. 26087377 10.1016/j.neuroimage.2015.06.022 PMC5553607

[R25] Fedyk M., Xu F. (2018). The epistemology of rational constructivism. *Review of Philosophy and Psychology*, 9(2), 343–62.

[R26] Frazier J.A., Chiu S., Breeze J.L., et al. (2005). Structural brain magnetic resonance imaging of limbic and thalamic volumes in pediatric bipolar disorder. *American Journal of Psychiatry*, 162(7), 1256–65. 10.1176/appi.ajp.162.7.1256 15994707

[R27] Gabard-Durnam L.J., Flannery J., Goff B., et al. (2014). The development of human amygdala functional connectivity at rest from 4 to 23years: a cross-sectional study. *Neuro Image*, 95, 193–207. 24662579 10.1016/j.neuroimage.2014.03.038 PMC4305511

[R28] Gee D.G., Humphreys K.L., Flannery J., et al. (2013). A developmental shift from positive to negative connectivity in human amygdala-prefrontal circuitry. *Journal of Neuroscience*, 33(10), 4584–93. 23467374 10.1523/JNEUROSCI.3446-12.2013 PMC3670947

[R29] Goldstein J.M., Seidman L.J., Makris N., et al. (2007). Hypothalamic abnormalities in schizophrenia: sex effects and genetic vulnerability. *Biological Psychiatry*, 61(8), 935–45. 17046727 10.1016/j.biopsych.2006.06.027

[R30] Greene D.J., Black K.J., Schlaggar B.L. (2016). Considerations for MRI study design and implementation in pediatric and clinical populations. *Developmental Cognitive Neuroscience*, 18, 101–12. 26754461 10.1016/j.dcn.2015.12.005 PMC4834255

[R31] Greene D.J., Laumann T.O., Dubis J.W., et al. (2014). Developmental changes in the organization of functional connections between the basal ganglia and cerebral cortex. *Journal of Neuroscience*, 34(17), 5842–54. 24760844 10.1523/JNEUROSCI.3069-13.2014 PMC3996213

[R32] Greene J.D. (2007). Why are VMPFC patients more utilitarian? A dual-process theory of moral judgment explains. *Trends in Cognitive Sciences*, 11(8), 322–3. 17625951 10.1016/j.tics.2007.06.004

[R33] Haxby J.V., Connolly A.C., Guntupalli J.S. (2014). Decoding neural representational spaces using multivariate pattern analysis. *Annual Review of Neuroscience*, 37, 435–56. 10.1146/annurev-neuro-062012-170325 25002277

[R34] Hayes A.F. (2020). SPSS, SAS and R macros and code. Available: http://afhayes.com/spss-sas-and-r-macros-and-code.html [February 15, 2020].

[R35] Hiser J., Koenigs M. (2018). The multifaceted role of the ventromedial prefrontal cortex in emotion, decision making, social cognition, and psychopathology. *Biological Psychiatry*, 83(8), 638–47. 29275839 10.1016/j.biopsych.2017.10.030 PMC5862740

[R36] Hoemann K., Xu F., Barrett L.F. (2019). Emotion words, emotion concepts, and emotional development in children: a constructionist hypothesis. *Developmental Psychology*, 55(9), 1830–49. 31464489 10.1037/dev0000686 PMC6716622

[R37] Jalbrzikowski M., Larsen B., Hallquist M.N., Foran W., Calabro F., Luna B. (2017). Development of white matter microstructure and intrinsic functional connectivity between the amygdala and ventromedial prefrontal cortex: associations with anxiety and depression. *Biological Psychiatry*, 82(7), 511–21. 28274468 10.1016/j.biopsych.2017.01.008 PMC5522367

[R38] Jenkinson M., Beckmann C.F., Behrens T.E.J., Woolrich M.W., Smith S.M. (2012). FSL. *Neuro Image*, 62(2), 782–90. 21979382 10.1016/j.neuroimage.2011.09.015

[R39] Karim H.T., Perlman S.B. (2017). Neurodevelopmental maturation as a function of irritable temperament. *Human Brain Mapping*, 38(10), 5307–21. 28737296 10.1002/hbm.23742 PMC5752122

[R40] Kassam K.S., Markey A.R., Cherkassky V.L., Loewenstein G., Just A. (2013). Identifying emotions on the basis of neural activation. *PLOS ONE*, 8(6), 12. 10.1371/journal.pone.0066032 PMC3686858 23840392

[R41] Knutson B., Greer S.M. (2008). Anticipatory affect: neural correlates and consequences for choice. *Philosophical Transactions of the Royal Society B: Biological Sciences*, 363(1511), 3771–86. 10.1098/rstb.2008.0155 PMC2607363 18829428

[R42] Kragel P.A., LaBar K.S. (2015). Multivariate neural biomarkers of emotional states are categorically distinct. *Social Cognitive and Affective Neuroscience*, 10(11), 1437–48. 25813790 10.1093/scan/nsv032 PMC4631142

[R43] Kragel P.A., LaBar K.S. (2016). Decoding the nature of emotion in the brain. *Trends in Cognitive Sciences*, 20(6), 444–55. 27133227 10.1016/j.tics.2016.03.011 PMC4875847

[R44] Kriegeskorte N., Mur M., Bandettini P. (2008). Representational similarity analysis—Connecting the branches of systems neuroscience. *Frontiers in Systems Neuroscience*, 2, 1–28. 19104670 10.3389/neuro.06.004.2008 PMC2605405

[R45] Krippendorff K. (2004). Reliability in content analysis: some common misconceptions and recommendations. *Human Communication Research*, 30(3), 411–33.

[R46] Krumhuber E.G. Manstead A.S.R. (2009). Can Duchenne smiles be feigned? New evidence on felt and false smiles. *Emotion*, 9(6), 807–20. 20001124 10.1037/a0017844

[R47] Larson R., Csikszentmihalyi M., Graef R. (1980). Mood variability and the psychosocial adjustment of adolescents. *Journal of Youth and Adolescence*, 9(6), 469–91. 24318310 10.1007/BF02089885

[R48] Levita L., Hare T.A., Voss H.U., Glover G., Ballon D.J., Casey B.J. (2009). The bivalent side of the nucleus accumbens. *Neuro Image*, 44(3), 1178–87. 18976715 10.1016/j.neuroimage.2008.09.039 PMC2659952

[R49] Lombard M. (2013). Lessons learned from a research saga: An ambitious content analysis of television form. In: Valdivia, A. N., editors. The International Encyclopedia of Media Studies, pp. 303–18, Malden, MA.

[R50] Lombard M., Snyder-Duch J., Bracken C.C. (2002). Content analysis in mass communication: assessment and reporting of intercoder reliability. *Human Communication Research*, 28(4), 587–604.

[R51] Ma X., Tamir M., Miyamoto Y. (2018). A socio-cultural instrumental approach to emotion regulation: culture and the regulation of positive emotions. *Emotion*, 18(1), 138–52. 28414476 10.1037/emo0000315

[R52] Makris N., Goldstein J.M., Kennedy D., et al. (2006). Decreased volume of left and total anterior insular lobule in schizophrenia. *Schizophrenia Research*, 83(2–3), 155–71. 16448806 10.1016/j.schres.2005.11.020

[R53] Moll J., de Oliveira-souza R. (2007). Moral judgments, emotions and the utilitarian brain. *Trends in Cognitive Sciences*, 11(8), 319–21. 17602852 10.1016/j.tics.2007.06.001

[R54] Monk C.S., Klein R.G., Telzer E.H., et al. (2008). Amygdala and nucleus accumbens activation to emotional facial expressions in children and adolescents at risk for major depression. *American Journal of Psychiatry*, 165(1), 90–8. 10.1176/appi.ajp.2007.06111917 17986682

[R55] Moses P., DiNino M., Hernandez L., Liu T.T. (2014). Developmental changes in resting and functional cerebral blood flow and their relationship to the BOLD response: CBF and BOLD responses in children. *Human Brain Mapping*, 35(7), 3188–98. 24142547 10.1002/hbm.22394 PMC6868989

[R56] Noftle E.E., Fleeson W. (2010). Age differences in big five behavior averages and variabilities across the adult life span: moving beyond retrospective, global summary accounts of personality. *Psychology and Aging*, 25(1), 95–107. 20230131 10.1037/a0018199 PMC2922931

[R57] Nook E.C., Sasse S.F., Lambert H.K., McLaughlin K.A., Somerville L.H. (2017). Increasing verbal knowledge mediates development of multidimensional emotion representations. *Nature Human Behaviour*, 1(12), 881–9. 10.1038/s41562-017-0238-7 PMC5790154 29399639

[R58] Nook E.C., Sasse S.F., Lambert H.K., McLaughlin K.A., Somerville L.H. (2018). The nonlinear development of emotion differentiation: granular emotional experience is low in adolescence. *Psychological Science*, 29(8), 1346–57. 29878880 10.1177/0956797618773357 PMC6088506

[R59] O’Toole M.S., Renna M.E., Elkjær E., Mikkelsen M.B., Mennin D.S. (2020). A systematic review and meta-analysis of the association between complexity of emotion experience and behavioral adaptation. *Emotion Review*, 12(1), 23–38.

[R60] Ortony A., Clore G.L., Collins A. (1988). *The Cognitive Structure of Emotions*, Cambridge, MA: Cambridge University Press. doi: 10.1017/CBO9780511571299

[R61] Perlman S.B., Pelphrey K.A. (2011). Developing connections for affective regulation: age-related changes in emotional brain connectivity. *Journal of Experimental Child Psychology*, 108(3), 607–20. 20971474 10.1016/j.jecp.2010.08.006 PMC3029468

[R62] Pons F., Lawson J., Harris P.L., de Rosnay M. (2003). Individual differences in children’s emotion understanding: effects of age and language. *Scandinavian Journal of Psychology*, 44(4), 347–53. 12887556 10.1111/1467-9450.00354

[R63] Pons F., Harris P.L., de Rosnay M. (2004). Emotion comprehension between 3 and 11 years: developmental periods and hierarchical organization. *European Journal of Developmental Psychology*, 1(2), 127–52.

[R64] Popal H.S., Wang Y., Olson I.R. (2019). A guide to representational similarity analysis for social neuroscience. *Social Cognitive and Affective Neuroscience*, 14(11), 1243–53. 31989169 10.1093/scan/nsz099 PMC7057283

[R65] Pratto F., John O.P. (1991). Automatic vigilance: the attention-grabbing power of negative social information. *Journal off Personality and Social Psychology*, 61(3), 380–291. 10.1037//0022-3514.61.3.380 1941510

[R66] Richards J.M., Plate R.C., Ernst M. (2013). A systematic review of fMRI reward paradigms used in studies of adolescents vs. adults: the impact of task design and implications for understanding neurodevelopment. *Neuroscience and Biobehavioral Reviews*, 37(5), 976–91. 23518270 10.1016/j.neubiorev.2013.03.004 PMC3809756

[R67] Roseman I.J. Smith C.S. (2001). Appraisal theory: overview, assumptions, varieties, controversies. In: Scherer, K.R., Schorr, A., Johnstone, T., editors. *Appraisal Processes in Emotion: Theories, Methods, Research*, pp. 3–19, Oxford University Press. Oxford, UK.

[R68] de Rosnay M., Harris P.L. (2002). Individual differences in children’s understanding of emotion: the roles of attachment and language. *Attachment & Human Development*, 4(1), 39–54. 12065029 10.1080/14616730210123139

[R69] Russell J.A. (1980). A circumplex model of affect. *Journal of Personality and Social Psychology*, 39(6), 1161–78.

[R70] Russell J.A. (2003). Core affect and the psychological construction of emotion. *Psychological Review*, 110(1), 145–72. 12529060 10.1037/0033-295x.110.1.145

[R71] Saarimäki H., Gotsopoulos A., Jääskeläinen I.P., et al. (2016). Discrete neural signatures of basic emotions. *Cerebral Cortex*, 26(6), 2563–73. 25924952 10.1093/cercor/bhv086

[R72] Sakaki M., Yoo H.J., Nga L., Lee T.-H., Thayer J.F., Mather M. (2016). Heart rate variability is associated with amygdala functional connectivity with MPFC across younger and older adults. *Neuro Image*, 139, 44–52. 27261160 10.1016/j.neuroimage.2016.05.076 PMC5133191

[R73] Salzman C.D., Paton J.J., Belova M.A., Morrison S.E. (2007). Flexible neural representations of value in the primate brain. *Annals of the New York Academy of Sciences*, 1121(1), 336–54. 17872400 10.1196/annals.1401.034 PMC2376754

[R74] Satpute A.B., Nook E.C., Narayanan S., Shu J., Weber J., Ochsner K.N. (2016). Emotions in “black and white” or shades of gray? How we think about emotion shapes our perception and neural representation of emotion. *Psychological Science*, 27(11), 1428–42. 27670663 10.1177/0956797616661555 PMC5111864

[R76] Silvers J.A., McRae K., Gabrieli J.D.E., Gross J.J., Remy K.A., Ochsner K.N. (2012). Age-related differences in emotional reactivity, regulation, and rejection sensitivity in adolescence. *Emotion*, 12(6), 1235–47. 22642356 10.1037/a0028297 PMC4131311

[R77] Silvers J.A., Insel C., Powers A., et al. (2017). VlPFC–vmPFC–amygdala interactions underlie age-related differences in cognitive regulation of emotion. *Cerebral Cortex*, 27(7), 3502–14. 27341851 10.1093/cercor/bhw073 PMC6059245

[R78] Szczurek L., Monin B., Gross J.J. (2012). The stranger effect: the rejection of affective deviants. *Psychological Science*, 23(10), 1105–11. 22961772 10.1177/0956797612445314

[R79] van den Bos W., van Dijk E., Crone E.A. (2012). Learning whom to trust in repeated social interactions: a developmental perspective. *Group Processes & Intergroup Relations*, 15(2), 243–56.

[R80] Wei L., Chen H., Wu G.-R. (2018). Structural covariance of the prefrontal-amygdala pathways associated with heart rate variability. *Frontiers in Human Neuroscience*, 12, 1–11. 29545744 10.3389/fnhum.2018.00002 PMC5838315

[R81] Wellman H.M., Harris P.L., Banerjee M., Sinclair A. (1995). Early understanding of emotion: evidence from natural language. *Cognition & Emotion*, 9(2–3), 117–49.

[R82] Widen S.C. (2013). Children’s interpretation of facial expressions: the long path from valence-based to specific discrete categories. *Emotion Review*, 5(1), 72–7.

[R83] Widen S.C., Russell J.A. (2003). A closer look at preschoolers’ freely produced labels for facial expressions. *Developmental Psychology*, 39(1), 114–28. 12518813 10.1037//0012-1649.39.1.114

[R84] Widen S.C., Russell J.A. (2008). Children acquire emotion categories gradually. *Cognitive Development*, 23(2), 291–312.

[R85] Widen S.C., Russell J.A. (2010). Differentiation in preschooler’s categories of emotion. *Emotion*, 10(5), 651–61. 21038948 10.1037/a0019005

[R86] Winecoff A., Clithero J.A., Carter R.M., Bergman S.R., Wang L., Huettel S.A. (2013). Ventromedial prefrontal cortex encodes emotional value. *Journal of Neuroscience*, 33(27), 11032–9. 23825408 10.1523/JNEUROSCI.4317-12.2013 PMC3718369

[R87] Yang X., Garcia K.M., Jung Y., Whitlow C.T., McRae K., Waugh C.E. (2018). VmPFC activation during a stressor predicts positive emotions during stress recovery. *Social Cognitive and Affective Neuroscience*, 13(3), 256–68. 29462404 10.1093/scan/nsy012 PMC5836276

